# Comment on “Electrolytes in Muscle Pain: Systematic Review and Meta-Analysis with Implications for TMD”

**DOI:** 10.1016/j.identj.2026.109599

**Published:** 2026-04-23

**Authors:** Swarupanjali Padhi, Prashant Ramdas Kokiwar, Janvi Patel, Ankita Kalra

**Affiliations:** Department of Pharmaceutics, Noida Institute of Engineering & Technology (Pharmacy Institute), Greater Noida, India; Department of Community Medicine, Malla Reddy Institute of Medical Sciences, Hyderabad, Telangana, India; Dr. D. Y. Patil Medical College, Hospital and Research Centre, Dr. D. Y. Patil Vidyapeeth (Deemed-to-be-University), Pimpri, Pune, Maharashtra, India; Faculty of Pharmaceutical Sciences, Graphic Era Hill University, Dehradun, India; Centre for Promotion of Research, Graphic Era Deemed University, Dehradun, India

Dear Editor,

We read with interest this systematic review and meta-analysis evaluating electrolyte supplementation for muscle cramps and myalgia, and its possible relevance to temporomandibular disorder myalgia.^1^ The article addresses an important translational question and concludes that magnesium has the strongest, albeit indirect, rationale for further study in TMD, with benefit in pregnancy-associated cramps but inconsistent findings in adult nocturnal or persistent leg cramps.

The authors appropriately acknowledge heterogeneity and indirectness, but several reporting issues merit clarification. The Methods state that PubMed/MEDLINE, Embase, and CENTRAL were searched, whereas the PRISMA diagram attributes all 75 retrieved records to PubMed and none to Embase or Cochrane. In addition, the Results state that 34 full texts were assessed, while the PRISMA figure shows 22. A supplementary table reporting database-specific yields, duplicate removal, and reasons for exclusion at full-text review would improve reproducibility and confidence in the review process.^1^

A related concern is study eligibility and quantitative consistency. Table 1 excludes observational studies, cirrhosis-related cramps, and multi-ingredient products in which the electrolyte effect cannot be isolated, yet the included studies comprise an observational pregnancy trial, a cirrhosis trial, and multielectrolyte beverages.^1^ The pooled pregnancy estimate is reported as a risk ratio of 1.35 for achieving at least 50% cramp reduction, yet the forest plot appears to place the pooled estimate on the opposite side of the line of no effect. The adult pooled mean difference is also reported with a confidence interval of 1.15 to 0.31, which is internally inconsistent. Providing extracted 2 × 2 data, outcome coding, and corrected forest plots would substantially strengthen interpretability.^1^

From a clinical perspective, the conclusion that magnesium is the most plausible translational candidate for TMD myalgia should remain cautious. Direct TMD evidence currently rests on a randomized trial of local magnesium sulphate injection and a recent network meta-analysis suggesting benefit for magnesium sulphate, but the latter explicitly rated this evidence as very low certainty.^2^^,^^3^ Conversely, a recent systematic review found no clear first-choice drug for temporomandibular pain, and a clinical practice guideline favoured multimodal nonsurgical care over routine pharmacologic escalation as first-line management.^4^^,^^5^ Together, these data support magnesium as a hypothesis-generating option rather than a practice-changing inference.

This review raises a worthwhile mechanistic hypothesis and may help frame future TMD trials. Clarifying the search yield, eligibility decisions, and meta-analytic coding would improve confidence in the findings. A more measured interpretation of translational relevance would also better align the article with current evidence and clinical practice.

## Author contributions

All authors contributed to the conception, drafting, critical revision, and final approval of the manuscript.

## Funding

No specific funding was received for this work.

## Conflict of interest

The author(s) declare that there are no conflicts of interest regarding the publication of this letter.
